# Transmembrane domain dependent inhibitory function of FcγRIIB

**DOI:** 10.1007/s13238-018-0509-8

**Published:** 2018-03-01

**Authors:** Junyi Wang, Zongyu Li, Liling Xu, Hengwen Yang, Wanli Liu

**Affiliations:** 1 MOE Key Laboratory of Protein Sciences, Collaborative Innovation Center for Diagnosis and Treatment of Infectious Diseases, School of Life Sciences, Tsinghua University, Beijing, 100084 China; 2 Ragon Institute of Massachusetts General Hospital, Massachusetts Institute of Technology, Harvard University, 400 Technology Square, Cambridge, MA 02139 USA; 3 The First Affiliate Hospital, Biomedical Translational Research Institute, Guangdong Province Key Laboratory of Molecular Immunology and Antibody Engineering, Jinan University, Guangzhou, 510632 China

**Keywords:** B cell, FcγRIIB, transmembrane domain, systemic lupus erythematosus, autoimmune disease

## Abstract

FcγRIIB, the only inhibitory IgG Fc receptor, functions to suppress the hyper-activation of immune cells. Numerous studies have illustrated its inhibitory function through the ITIM motif in the cytoplasmic tail of FcγRIIB. However, later studies revealed that in addition to the ITIM, the transmembrane (TM) domain of FcγRIIB is also indispensable for its inhibitory function. Indeed, recent epidemiological studies revealed that a non-synonymous single nucleotide polymorphism (rs1050501) within the TM domain of FcγRIIB, responsible for the I232T substitution, is associated with the susceptibility to systemic lupus erythematosus (SLE). In this review, we will summarize these epidemiological and functional studies of FcγRIIB-I232T in the past few years, and will further discuss the mechanisms accounting for the functional loss of FcγRIIB-I232T. Our review will help the reader gain a deeper understanding of the importance of the TM domain in mediating the inhibitory function of FcγRIIB and may provide insights to a new therapeutic target for the associated diseases.

## INTRODUCTION

Receptors for the Fc portion of IgG molecules (FcγRs) play important roles in regulating the activation of immune cells for the purpose of balancing immunoprotection and immunopathology. The human immune system contains six types of canonical FcγRs, including the high affinity receptor FcγRI, and low affinity receptors FcγRIIA, FcγRIIB, FcγRIIC, FcγRIIIA, as well as FcγRIIIB, among which FcγRIIB is the only one possessing an inhibitory function (Amigorena et al., 1989; Nimmerjahn and Ravetch, 2011; Pincetic et al., 2014b; Smith and Clatworthy, 2010b). Activating FcγRs transduces a signal through the immunoreceptor tyrosine-based activation motif (ITAM) in its intracellular domain, or the associated signaling subunit called FcR common γ-chain (Blank et al., 2009; Daeron, 1997). In marked contrast, FcγRIIB possesses an immunoreceptor tyrosine-based inhibition motif (ITIM) in its intracellular domain (Bolland and Ravetch, 2000). When activated by antigen-antibody immune complex, the tyrosine within the ITIM could be phosphorylated by the Src-family kinase Lyn, leading to recruitment of SH2-containing SHIP and/or SHP to transduce inhibitory signal cascades (Nimmerjahn and Ravetch, 2008). FcγRIIB is ubiquitously expressed on most types of immune cells including dendritic cells (DCs), monocytes, macrophages, mast cells, neutrophils, basophils and even memory CD8^+^ T cells (Daeron et al., 1993; Ravetch and Kinet, 1991; Starbeck-Miller et al., 2014). In addition, FcγRIIB was found to be expressed in microglia, participating in the phagocytosis in central nervous system (CNS) infection (Peress, 1993). In B cells, FcγRIIB serves as the exclusive FcγR expressed on the plasma membrane, which potently suppresses B cell activation, antigen internalization and presentation to T cells after binding to the antigen-antibody complex. All of these studies reinforce the universal and crucial role of FcγRIIB in down-regulating the activation and function of all these immune cells (Lehmann et al., 2012). Thus, malfunction of FcγRIIB is usually detrimental for the immune system (Niederer et al., 2010a; Pincetic et al., 2014a; Smith and Clatworthy, 2010a). Indeed, single nucleotide polymorphisms (SNPs) of the human FcγRIIB gene significantly influence the susceptibility to autoimmune diseases (Kyogoku et al., 2002b; Niederer et al., 2010a; Smith and Clatworthy, 2010a). Recent epidemiological studies revealed a non-synonymous single nucleotide polymorphism (rs1050501) within the transmembrane (TM) domain of FcγRIIB, causing the I232T substitution, is associated with the susceptibility to systemic lupus erythematosus (SLE) (Kyogoku et al., 2002a). In this review, we will summarize these epidemiological and functional studies of FcγRIIB-I232T in the past few years and will further discuss the mechanisms accounting for the functional loss of FcγRIIB-I232T. We will also discuss the evolutionary conservation of the TM domain among different FcγRs in different species. Our review will help the reader gain a deeper understanding of the importance of the TM domain in mediating the inhibitory function of FcγRIIB and may provide insights to a new therapeutic target for the associated diseases.

## EPIDEMIOLOGY STUDIES OF FcγRIIB-I232T WITH AUTOIMMUNE DISEASES

Human *FCGR2B* gene possesses a number of SNPs, including several SNPs in the promoter region and eight missense SNPs. Amongst the nonsynonymous SNPs, the T-to-C transition in exon 5 (rs1050501), which leads to a replacement of isoleucine at position 232 by threonine (FcγRIIB-I232T variant), occurs at a notable frequency and is associated with autoimmune disease. In 2002, Kyogoku first demonstrated the correlation of FcγRIIB-I232T with the susceptibility to SLE in Japanese (Kyogoku et al., 2002a), while the epidemiology studies of FcγRIIB-I232T in the context of autoimmune diseases sprung up in the years to follow. Later studies further confirmed that FcγRIIB-I232T was also associated with the susceptibility to SLE in Thai (Siriboonrit et al., 2003), Chinese (Chu et al., 2004) and also Caucasian (Willcocks et al., 2010) populations. According to Chu’s work, FcγRIIB-I232T is highly associated with nephritis in SLE patients (Chu et al., 2004). Interestingly, it is also found that homozygosity for this polymorphism is associated with protection against severe malaria, where selection pressure may contribute to the higher frequency of FcγRIIB-T232 and, hence, SLE in Africans and Southeast Asians (Willcocks et al., 2010).

Aside from SLE, rheumatoid arthritis (RA) is another common systemic autoimmune disease. Although epidemiology studies in literature did not find a significant correlation between FcγRIIB-I232T homozygotes with RA (Chen et al., 2008; Kyogoku et al., 2002c; Radstake et al., 2006), it is striking to observe that RA patients carrying homozygous FcγRIIB-T232 would develop severe radiologic joint damage during the first six-year course of the disease (Radstake et al., 2006). Besides, the FcγRIIB-I232T is associated with susceptibility to RA in Chinese population according to our unpublished statistical data.

Though epidemiological studies revealed the association of FcγRIIB-I232T with SLE and RA, the correlation studies with other autoimmune diseases are still scarce. It has already been proved that there is a lower expression level of FcγRIIB in multiple sclerosis (MS) patients (Tackenberg et al., 2009). And among idiopathic thrombocytopenic purpura (ITP) patients, a higher frequency of hetero FcγRIIB-I232/I232T genotype is observed in chronic disease patients compared to that in acute disease patients (Bruin et al., 2004). All these results indicate that FcγRIIB may play a sophisticated role in different autoimmune diseases.

## TRANSMEMBRANE DOMAIN-DEPENDENT INHIBITORY FUNCTION OF FcγRIIB

Although numerous studies have reported the critical role of ITIM as a prerequisite for the inhibitory function of FcγRIIB, recent reports also revealed that the unique property of the TM domain is also essential for its role of inhibition. In this section, we will discuss the four possible mechanisms through which the TM-domain is required for the inhibitory function of FcγRIIB: (1) impeding the conformational changes of BCR, which is crucial for the initiation of B cell receptor (BCR) downstream signaling; (2) co-localizing with lipid raft to activate the inhibitory signaling; (3) blocking the synaptic co-localization of BCR and CD19 microclusters; (4) conferring fast lateral mobility.

**Figure 1:**
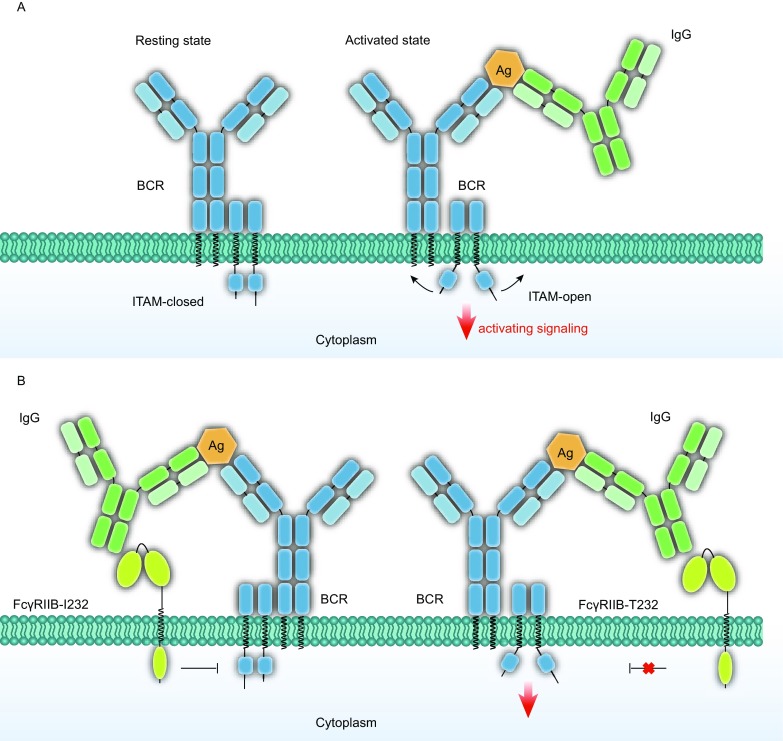
**A conformational change blocking model**. (A) Shown are the “close to open” conformational changes within the cytoplasmic tail of BCR during the cross-membrane signal transduction. (B) FcγRIIB-I232 blocks such conformational changes in the cytoplasmic domains of BCR, whereas FcγRIIB-T232 fails to do so

The initiation of BCR signaling is reported to be accompanied by a “closed to open” form transition of the cytoplasmic domains of the BCR (Sohn et al., 2008b; Tolar et al., 2005) (Fig. 1A). According to model 1, blocking the conformational changes in the cytoplasmic domains of the BCRs might be one of the approaches for FcγRIIB to play its inhibitory role. Indeed, FcγRIIB was proved to block the transition to active signaling conformation when colligated with BCR, however FcγRIIB-I232T failed to inhibit such conformational changes of BCR (Liu et al., 2010), even though FcγRIIB-I232T maintains an intact extracellular domain for ligand recognition and an intact intracellular ITIM (Fig. 1B).

**Figure 2:**
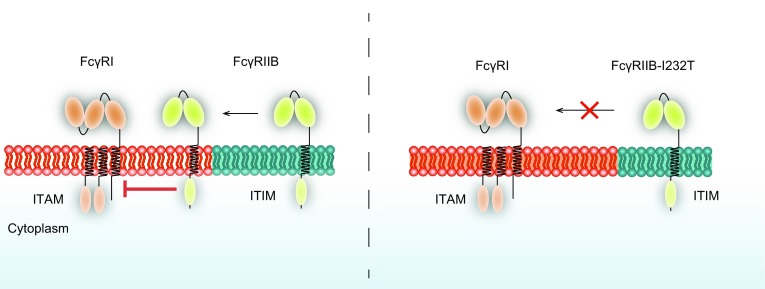
**A diagram of the lipid raft-residing model explaining the dysfunction of FcγRIIB-I232T**. Model of the FcγRIIB and FcγRIIB-I232T showing that FcγRIIB-I232T fails to distribute to lipid raft, which results in the impairment of the inhibitory function. Orange color shows lipid raft

It was indicated by fluorescence resonance energy transfer (FRET) assay that the interaction between BCR and lipid raft transiently take place at the periphery of the B cell immunological synapse where BCR-antigen clusters are newly formed (Sohn et al., 2008b). In contrast, the interaction between FcγRIIB and lipid raft is much more stable and the co-localization of FcγRIIB with raft lipid is found to be independent of the activity of Src family kinase (Liu et al., 2010). In model 2, when co-localizing with BCR in the lipid raft, FcγRIIB inhibits the early events of BCR activation, such as BCR recruitment and oligomerization in the lipid raft, in addition to BCR downstream signaling. In 2002, Kono et al. indicated that spatial association between the FcγRIIB TM domain and lipid raft is essential for its inhibitory signaling (Kono et al., 2002). However, FcγRIIB-I232T exhibited defective membrane proximal signaling compared to FcγRIIB-WT (Floto et al., 2005). The subsequent study by Western blot and live cell imaging with fluorescently labeled cholera toxin B, a lipid raft marker, discovered more FcγRIIB co-localized with lipid raft than FcγRIIB-I232T, confirming the necessity of the residence to sphingolipid rafts (Floto et al., 2005; Kono et al., 2005). As the signal pathway dependent on the interaction between FcγRIIB-I232T and protein kinases that reside preferentially in lipid-raft-rich domains might be interfered, thus the inhibitory function is impaired (Fig. 2).

**Figure 3:**
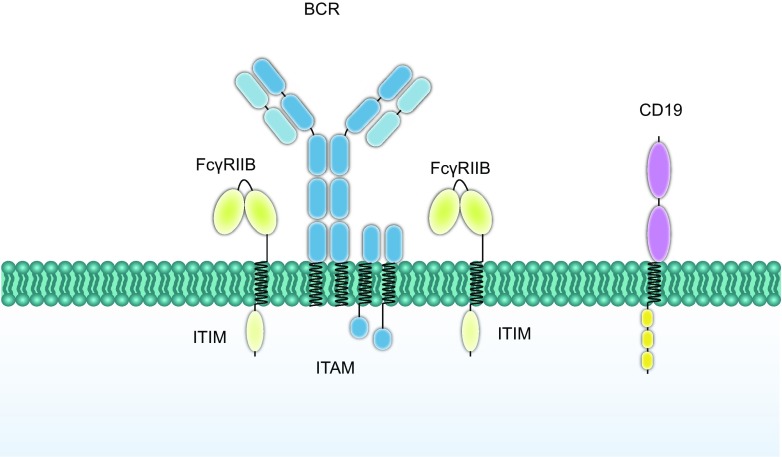
**A CD19-blocking model that reveals the inhibitory function of FcγRIIB**. The FcγRIIB can inhibit B cell activation by preventing CD19, which is an activating co-receptor, from colocalizing with BCR microclusters. The impaired function of FcγRIIB-I232T can also be explained as the failure in blocking the colocalization

Model 3 relies on the finding that the TM domain of FcγRIIB is responsible for blocking the synaptic colocalization of BCR and CD19 microclusters. CD19 is the activating co-receptor of BCR, which promotes BCR signaling when co-localizing with BCR in the lipid raft during B cell activation. In 2000, Fong et al. noticed that dephosphorylation of CD19 was independent of the cytoplasmic tail of FcγRIIB (Fong et al., 2000). Our study showed that both of FcγRIIB with or without cytoplasmic tail could significantly impair the colocalization of BCR and CD19 microclusters, however a chimeric construct with a truncated TM from fruit fly N-Cadherin completely lost this inhibitory function (Xu et al., 2014). The loss-of-function mutant FcγRIIB-I232T also failed to block the synaptic colocalization of the BCR with CD19, leading to dysregulated recruitment of downstream signaling molecule pPI3K to the membrane proximal signalosome. Early studies provided evidence that FcγRIIB-I232T is incapable of inhibiting receptor activation due to the reduced affinity to sphingolipid rafts, whereas strikingly, the chimeric construct containing a truncated TM of a lipid rafts resident protein, linker for activation of T cells (LAT) (Tanimura et al., 2003) could not rescue such inhibitory function (Xu et al., 2014). This result indicated that the inhibitory function of blocking synaptic colocalization of BCR and CD19 microclusters may depend on its unique TM sequence instead of its affinity to lipid rafts. Therefore, a possible functional model is that FcγRIIB prevents the colocalization of BCR and CD19 to attenuate the boosting of BCR signaling from CD19 (Fig. 3).

**Figure 4:**
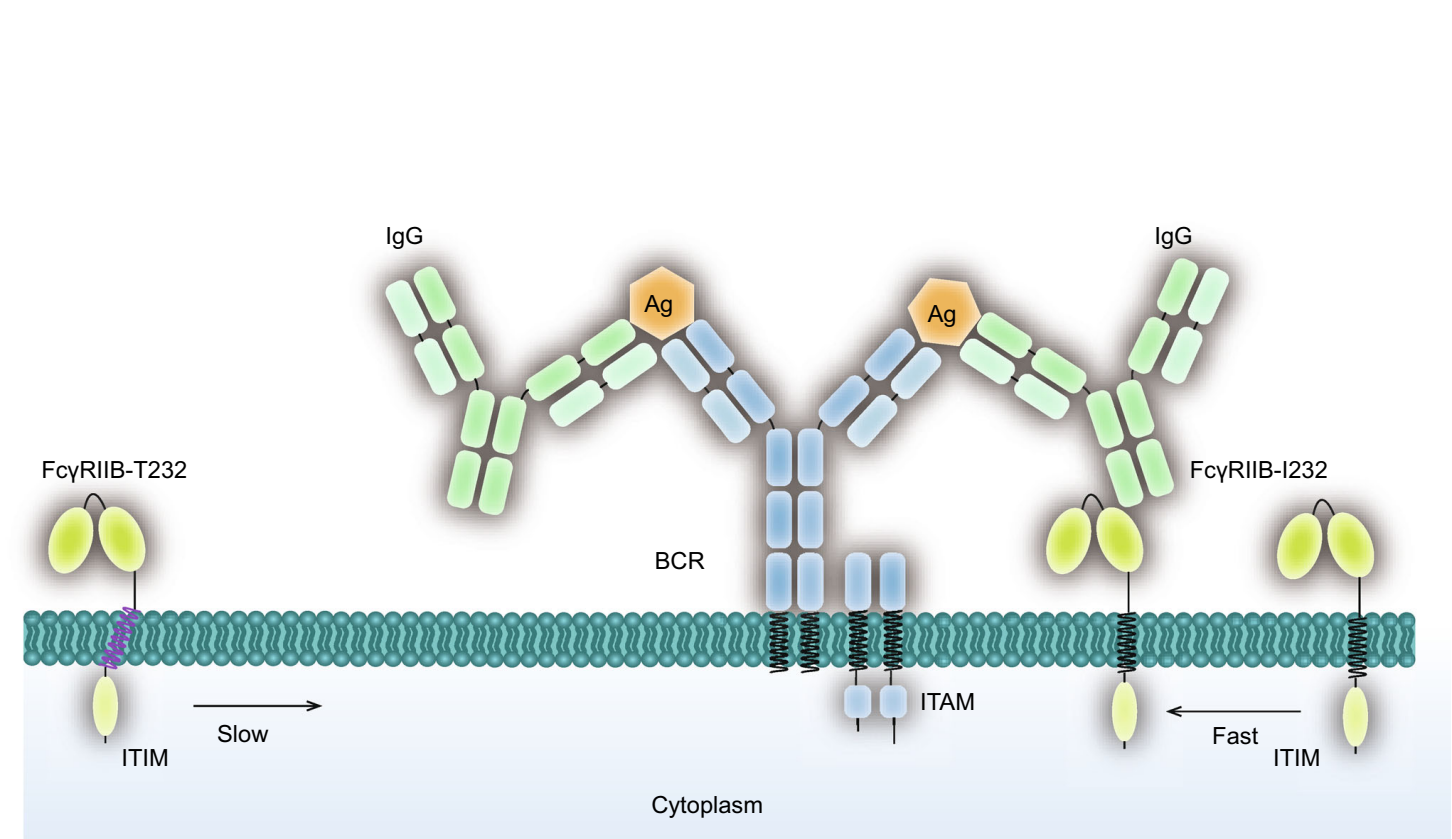
**The “catch-me-if-you-can” model**. The model indicates that the fast lateral mobility of FcγRIIB is crucial for its inhibitory function. Due to the high affinity between BCR and antigen, the BCR is more likely to catch the antigen before FcγRIIB catch the IgG upon immunocomplex stimulation. Then the low affinity FcγRIIB-WT has to diffuse fast enough to catch the IgG and stop the BCR signaling in time. In marked contrast, FcγRIIB-I232T fails to “catch” the IC due to its slow diffusion and thus loses its inhibitory function

Recent studies also revealed the polymorphism FcγRIIB-I232T affects the lateral mobility of FcγRIIB, and its enrichment to the immune complexes (ICs) significantly decreases after stimulation. However, if FcγRIIB-I232T was given sufficient time to diffuse and interact with ICs or its TM domain was substituted with one of a fast floating CD86 molecule, its inhibitory function can be reinstated (Xu et al., 2016). Hence, the lateral mobility could be one of the key factors for FcγRIIB to downregulate the BCR signaling. This mechanistic explanation of how a single amino acid change in the TM domain of FcγRIIB results in its dysfunction, which can be described as a “catch-me-if-you-can” model (model 4, Fig. 4). This model suggests that the inhibitory function of FcγRIIB is dependent on its lateral mobility, which is a property mainly determined by the TM domain of the receptor. It is generally believed that FcγRIIB acts mainly as a co-receptor to regulate the activation of the main activating immune receptors like FcγRI and BCR, which show high affinity to their ligand at nanomole range (Batista and Neuberger, 2000; Lu et al., 2015). In contrast, FcγRIIB and its ligand, Fc portion of human IgG antibody, show low-affinity at micromole range (Mimura et al., 2001). In this case, it is very likely that BCR binding to antigens is much more instantaneous than FcγRIIB colliding with the Fc of IgG upon IC stimulation. BCR is a relatively slow diffusion molecule, which becomes nearly immobile after encountering antigens. Defeated in the initial binding competition, FcγRIIB has to diffuse in a random manner until it collides with the Fc portion of clustered IgG with avidity effects in preformed IC microclusters, which finally enriches these molecules to initiate their inhibitory function. In order to collide with the Fc region of IgG in the immune-complex that has been caught by the BCRs, the faster that FcγRIIB diffuses, the better of a chance it would have at catching the Fc region and induce inhibition to the activation signaling from BCR in a timely manner. The model is also supported by the study using the PALM-based super-resolution imaging and Monte Carlo simulation analysis, which suggested that the lack of sufficient lateral mobility of FcγRIIB-I232T hinders its successful collision and enrichment in the IC microclusters and the subsequent assembly of larger-sized FcγRIIB nanoclusters in response to ICs (Xu et al., 2016).

## UNIQUE SEQUENCE OF FcγRIIB TM DOMAIN

**Figure 5:**
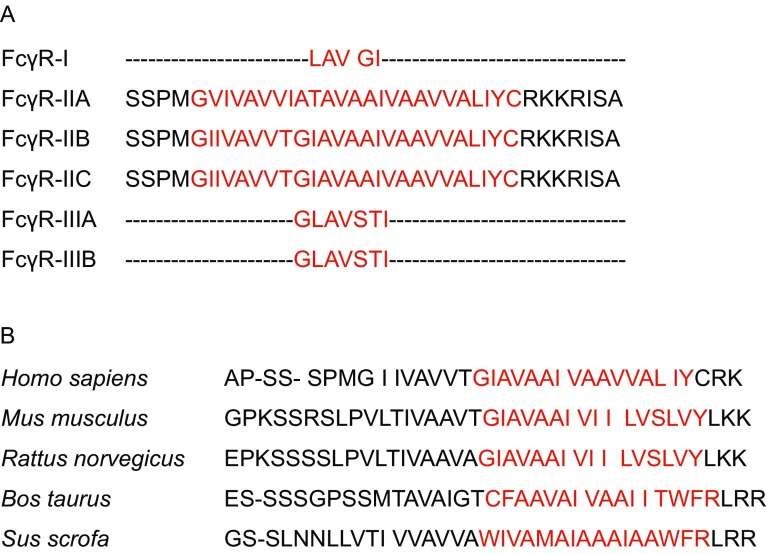
**An alignment of the transmembrane domain of Fcγ receptors**. (A) Shown are alignments of the transmembrane domain sequences of low affinity Fcγ receptors in NCBI. The conserved regions are highlighted in red color. (B) Shown are alignments of the transmembrane domain sequences from different species that are available in NCBI. The conserved regions are highlighted in red color

The TM domain sequences of human FcγRII members are highly conserved among different subclasses but differing from that of FcγRI and FcγRIII (Fig. 5A). Amongst the FcγRII family, FcγRIIC, which has the same extracellular and TM domains as FcγRIIB and the same cytoplasmic tail as FcγRIIA, is believed to generate from the gene recombination between FcγRIIB and FcγRIIA (Warmerdam et al., 1993). The SNP Q57stop in FcγRIIC, up-regulating the expression of this activating FcγRIIC, leads to enhanced immune response and autoimmune disease in both human patients and transgenic mice (Li et al., 2013). Different, but analogous to FcγRIIB, which possesses Thr230 and Ile232, FcγRIIA has a Thr at site 232 and an Ile at site 230 (Fig. 5A), however, thus far no study has reported any dysfunction of this TM domain. Notably, protein sequences of the TM domain of FcγRIIB are evolutionarily conserved amongst species (Fig. 5B). All functional FcγRIIBs conserve the Thr residue in the TM domain, however, it is remarkable that none of them has two Thr distributed in such close positions like FcγRIIB-I232T. The substitution of Ile to Thr at site 232 inserts a polar amino acid residue into the TM domain, which definitely bends the α-helices of the TM domain (Ballesteros et al., 2000). This was further supported by the molecular dynamics simulation results which suggested that the bending of the FcγRIIB TM helix can be exacerbated by the residue substitution of I232T (Xu et al., 2016). Thus, the appearance of Thr230 and Thr232 at the TM domain of FcγRIIB-I232T in such close proximity may cause an unbearable bending, which would lead to a decreased lateral mobility.

Indeed, when we swapped the TM domain of FcγRIIB-I232T into different type-I TM molecules, such as TfR, MHC-1 and CD86 (Xu et al., 2014), we found only TM-CD86 could rescue the inhibitory function like wild type FcγRIIB (Xu et al., 2016). Among them, TfR was shown to be a non-lipid-raft associated protein (Harder et al., 1998) and MHC-1 had a slow lateral mobility (Georgiou et al., 2002), while CD86 had the shortest predicted TM domain and fastest lateral mobility (Xu et al., 2016). As expected, the chimeric constructs FcγRIIB-TM-TfR showed a loss-of-function phenomenon (Xu et al., 2014). This result combined with the result of FcγRIIB-TM-LAT suggests that a sole non-lipid raft residing or constitutive lipid raft residing TM cannot rescue the inhibitory function of FcγRIIB. There could be two explanations for this result according to the four models above: First, there was an early report by Sohn et al. showing that FcγRIIB is not located in lipid raft regions in quiescent B cells, but translocated to the lipid rafts upon BCR and FcγRIIB co-ligation (Sohn et al., 2008a). Second, the sequence of the TM domain of FcγRIIB is also important in exerting its inhibitory function. It is generally believed that FcγRIIB acts mainly as a co-receptor to regulate the activation of the main activating immune receptors like FcγRI and BCR, which show high affinity to their ligand at nanomole range (Batista and Neuberger, 2000; Lu et al., 2015). Comparatively, FcγRIIB and its ligand, Fc portion of human IgG antibody, show low-affinity at micromole range (Mimura et al., 2001). In this case, it is very likely that BCR binding to antigens is much more instantaneous than FcγRIIB colliding with the Fc of IgG upon immunocomplex stimulation.

## CONCLUSION AND FUTURE DIRECTION

The association between FcγRIIB-I232T and autoimmune diseases has been identified for over 14 years. Lots of studies have revealed the correlation, but the mechanism by which I232T substitution in the TM domain causes the loss-of-function phenotype is not entirely known yet. A better understanding of this SNP shall give insight to the subtle regulation of the TM domain of type-I transmembrane receptor to their functions and may provide a new approach and target for autoimmune disease diagnosis and therapies.

A recent study revealed that the inhibitory function of FcγRIIB is dependent on its TM domain, rather than the ITIM motif present in the cytoplasmic domain, to block the synaptic colocalization of BCR and CD19 microclusters (Xu et al., 2014). We propose that the TM domain provides an extra layer of inhibitory function for FcγRIIB to secure its unique position as the only inhibitory IgG FcR to downregulate immune responses (Hippen et al., 1997). Although the exact molecular mechanism remains unclear, it is revealed that the proper TM domain of FcγRIIB can impede BCR’s conformational changes, activate the inhibitory signaling distributed to lipid rafts, inhibit the recruitment of CD19 to B cell IS, which depends on its fast lateral mobility. The importance of the FcγRIIB TM domain is also supported by a rich amount of epidemiological studies showing a strong positive correlation of the FcγRIIB-I232T mutant with SLE disease (Chen et al., 2006; Chu et al., 2004; Clatworthy et al., 2007; Kyogoku et al., 2002a; Kyogoku et al., 2004; Niederer et al., 2010a; Niederer et al., 2010b; Pan et al., 2006; Siriboonrit et al., 2003; Willcocks et al., 2010).

In conclusion, the inhibitory role of the FcγRIIB TM domain in BCR signaling may functionally explain the pathological mechanism of SLE and other autoimmune diseases.
